# Histopathological aspects of the inclusion of surgical material in micrographic surgery using the Munich method and its comparison with horizontal histological sections^☆^^☆☆^

**DOI:** 10.1016/j.abd.2020.04.001

**Published:** 2020-05-16

**Authors:** Airá Novello Vilar, Arthur César Farah Ferreira

**Affiliations:** Private Clinic, Concórdia, SC, Brazil

Dear Editor,

The Munich method for micrographic surgery is technically distinct from the Mohs technique, both regarding the surgery itself and laboratory processing, as well as the microscopic analysis. The surgical specimen is usually examined without division, as long as its size allows for full inclusion.^1^

In the Munich technique, originally described in 1992 and published in Germany in 1995, the surgical specimen is frozen, usually outside the cryostat, by a direct stream of CO_2_ and with the use of distilled water, and then inserted in the cryostat to be sliced.^2^ However, we have, similarly to other colleagues, frozen the specimen directly in the cryostat with the use of OCT, as is customary in the intraoperative technique not only for skin, but for several other tissues.^3^, ^4^

Presented as a “new way of assessing debulking,” from the technical and laboratory standpoint, the method described by Portela et al.^5^ with horizontal sections, is identical to the Munich technique, despite starting from the surface to the depth and the fact that the interval and the thickness of sections are different, which may vary due to the peculiarities of each tissue. Likewise, the observation of the tumor and its relationship with the surgical margins is one of the most striking features of the Munich method.

Moreover, the aforementioned authors confuse the surgical margin with the surgical border, stating that the Mohs method, which is peripheral, examines the surgical margin and not the hypothetical surgical border (*i.e*., the section that is deposited on the microscope slide after the sectioning of the block).

While perhaps not identical, the Munich technique should at least have been referred to by the authors as the original idea, since it has been widely described in the literature, including in Anais Brasileiros de Dermatologia.

## Financial support

None declared.

## Authors' contributions

Airá Novello Vilar: Approval of the final version of the manuscript; conception and planning of the study; drafting and editing of the manuscript.

Arthur César Farah Ferreira: Critical review of the literature; critical review of the manuscript.

## Conflicts of interest

None declared.

## References

[1] Kopke L.F.F., Konz B. (1994). The fundamental differences among the variations of micrografic surgery. An Bras Dermatol.

[2] Kopke L.F.F., Konz B. (1995). Mikrographische Chirurgie. Eine methodische Bestandsaufnahme. Hautarzt.

[3] Davis D.A., Pellowski D.M., Hanke C.W. (2004). Preparation of frozen sections. Dermatol Surg.

[4] Dogan M.M., Snow S.N., Lo J. (1991). Rapid skin edge elevation using the OCT compound droplet technique to obtain horizontal microsections in Mohs micrographic surgery. J Dermatol Surg Oncol.

[5] Portela P.S., Teixeira D.A., Machado C.D.A.S., Pinhal M.A.S., Paschoal F.M. (2019). Horizontal histological sections in the preliminary evaluation of basal cell carcinoma submitted to Mohs micrographic surgery. An Bras Dermatol.

